# Use of financial incentives and text message feedback to increase healthy food purchases in a grocery store cash back program: a randomized controlled trial

**DOI:** 10.1186/s12889-019-6936-5

**Published:** 2019-05-31

**Authors:** Anjali Gopalan, Pamela A. Shaw, Raymond Lim, Jithen Paramanund, Deepak Patel, Jingsan Zhu, Kevin G. Volpp, Alison M. Buttenheim

**Affiliations:** 1 Division of Research, Kaiser Permanente Northern California Division of Research, 2000 Broadway, Oakland, CA94612 USA; 2 Department of Biostatistics, Epidemiology, and Informatics, Perelman School of Medicine, University of Pennsylvania, Philadelphia, PA USA; 3 Center for Health Incentives and Behavioral Economics, University of Pennsylvania, Philadelphia, PA USA; 4 Departments of Medicine and Medical Ethics and Health Policy, Perelman School of Medicine, University of Pennsylvania, Philadelphia, PA USA; 5 Discovery Vitality, Johannesburg, South Africa; 6 Department of Family and Community Health, University of Pennsylvania School of Nursing, Philadelphia, PA USA

**Keywords:** Diet, Nutrition, Financial incentives, Messaging

## Abstract

**Background:**

The HealthyFood (HF) program offers members up to 25% cash back monthly on healthy food purchases. In this randomized controlled trial, we tested the efficacy of financial incentives combined with text messages in increasing healthy food purchases among HF members.

**Methods:**

Members receiving the lowest (10%) cash back level were randomized to one of six arms: Arm 1 (Usual Care): 10% cash back, no weekly text, standard monthly text; Arm 2: 10% cash back, generic weekly text, standard monthly text; Arm 3: 10% cash back, personalized weekly text, standard monthly text; Arm 4: 25% cash back, personalized weekly text, standard monthly text; Arm 5: 10 + 15%NET cash back, personalized weekly text, standard monthly text; and, Arm 6: 10 + 15%NET cash back, personalized weekly text, unbundled monthly text. In the 10 + 15%NET cash back, the cash back amount was the baseline 10% plus 15% of the net difference between healthy and unhealthy spending. The generic text included information on HF and healthy eating, while the personalized text had individualized feedback on purchases. The standard monthly text contained the cash back amount. The unbundled monthly text included the amount lost due to unhealthy purchases. The primary outcome was the average monthly percent healthy food spending. Secondary outcomes were the percent unhealthy food spending, and the percent healthy and unhealthy food items.

**Results:**

Of the members contacted, 20 opted out, and 2841 met all inclusion criteria. There were no between-arm differences in the examined outcomes. The largest mean (standard deviation) difference in percent healthy spending was between Arm 1 (24.8% [11%]) and Arm 2 (26.8% [13%]), and the largest mean difference in percent unhealthy spending was also between Arm 1 (24.4% [20%]) and Arm 2 (21.7% [17%]), but no differences were statistically significant after correction for multiple comparisons.

**Conclusions:**

None of the tested financial incentive structures or text strategies differentially affected food purchasing. Notably, more than doubling the cash back amount and introducing a financial disincentive for unhealthy purchases did not affect purchasing. These findings speak to the difficulty of changing shopping habits and to the need for innovative strategies to shift complex health behaviors.

**Trial registration:**

NCT02486588 Increasing Engagement with a Healthy Food Benefit. The trial was prospectively registered on July 1, 2015.

**Electronic supplementary material:**

The online version of this article (10.1186/s12889-019-6936-5) contains supplementary material, which is available to authorized users.

## Background

Maintaining a healthy diet poses a considerable challenge for many people. There are myriad complex social, ecological, and psychological barriers to maintaining a healthy diet, including the higher cost of healthy foods, inadequate or incorrect nutritional knowledge, the enjoyment derived from less healthy foods, and a natural bias towards the present and short-term payoffs (e.g., the convenience of fast food) over potential long-term health benefits [1–4]. Past studies have demonstrated that financial savings can promote healthier food choices [5, 6]. Randomized interventions have demonstrated that participants who receive incentives, discounts or vouchers for healthy food items purchase greater quantities of fruits and vegetables [7, 8]. Prior work also supports the ability of tailored feedback to increase healthy eating behaviors; compared to generic information and messages, tailored nutrition-related messaging has a greater beneficial impact on individuals’ dietary behaviors [9, 10]. Currently lacking is information on how financial incentives and tailored messaging strategies can be combined to best promote healthier food choices during grocery shopping trips.

Discovery is a South Africa-based health insurance provider serving over 2.6 million members. Available to all Discovery members is the Vitality wellness program, a voluntary, low cost (329 South African Rand [R][≈ £18] per family/year) incentivized health promotion program. One of the largest programs is the HealthyFood benefit (HF), a three-tiered incentive program offering monthly cash back payments (up to max of R1000 [≈£55]) to incentivize the purchase of healthier food items at partner grocery chains. To allow Vitality to determine cash back payments, HF members have a membership card or linked credit card that when swiped during checkout at the partner grocers transmits purchase details to Vitality. Upon HF activation, members are eligible for 10% cash back on their healthy food expenditures at participating grocery stores (e.g., R10 cash back received if R100 spent on healthy foods during the month). “Healthy foods” include most fresh and frozen fruits and vegetables, low-fat dairy, whole grains, legumes, seeds, nuts, and selected oils (full catalog available at https://bit.ly/2MDHtXJ). This cash back percentage can increase to 15% upon the completion of an online health questionnaire and from 15 to 25% with completion of an in-person wellness check (i.e., blood pressure measurement, diabetes screening). As it stands, HF members have no financial disincentive for purchasing unhealthy foods and only receive feedback on their purchasing behaviors via a small notation on shopping receipts and through monthly cash back deposit notifications. Past work (analyses of member surveys and grocery scanner data) has demonstrated that HF enrollment and HF cash back amount are associated with healthier food purchasing [11, 12]. These results sparked interest in further exploring and rigorously testing whether changes to the HF benefit design could make the program more salient or motivating to members who may be less engaged with the current program and increase the healthiness of their food purchasing.

The purpose of this randomized controlled trial (RCT) was to test the effectiveness of differing financial incentive structures and text messaging feedback strategies in increasing healthy food purchases amongst HF members. We hypothesized that two strategies, increasing the salience of the financial incentive earned for healthy food purchases by increasing its size and highlighting the financial losses incurred from a new disincentive for unhealthy food purchases, would most effectively shift participants’ food purchasing.

## Methods

### Study design

The protocol was approved by the University of Witwatersrand Ethics Committee (Johannesburg, South Africa) and designated as exempt by the University of Pennsylvania Institutional Review Board. Potentially eligible members were identified by the Vitality team usng their member databases. While no formal consent process was required given existing language in the Vitality membership agreement, all individuals were given an opportunity to opt out of participation prior to randomization. The study biostatistician generated a randomization list that was sent to the Vitality team, who then linked it with the member database by study ID. Using a simple randomization scheme, members were assigned to one of the six study arms with equal probability. Following randomization, all members who did not opt out were sent an email describing and providing examples of any changes they would experience with their HF program during the intervention period. All operational aspects of the study (e.g., sending text messages and emails, administering monthly cash back payments) were performed by the Vitality team. The analytic team had no contact with participating HF members and was blinded to study arm assignment. Since this study addressed differing financial incentives and text messages by study arm, participant blinding after randomization was not feasible.

### Study population

Eligible individuals were adult Vitality members who activated the HF benefit in 2014 but had remained at the baseline 10% cash back level through the beginning of October 2015 (i.e., likely less engaged as they had not completed the tasks to move to a higher cash back amount). At the time of HF program enrollment, individuals chose one of two available grocery store chain partners as their preferred grocer. While members monthly cash back is determined based on healthy food spending at both partner grocers (i.e., not just the member’s preferred grocer), to accommodate our rapid feedback message design, we focused on individuals who had chosen a specific large grocery store chain as their preferred grocery store, as that grocer had the shortest lag between purchases and data transfer to Vitality. To ensure that the intervention targeted members who regularly shopped at this selected grocer (vs. made occasional small purchases there), we screened participants for a minimum of R1000 (≈£55) spent on groceries in the month prior to randomization, with at least 90% of this spending at this selected grocer. Finally, given the nature of the enrollment process and the tested interventions, we excluded those without an available email address and mobile phone number.

### Study outcomes

In the HF program, all food items are categorized as healthy, neutral, or unhealthy. Healthy foods are marked with a Vitality sticker (on shelves) and noted on shopping receipts. The primary study outcome was the average monthly percent healthy food spending at the selected grocer during the Full Intervention period. Monthly percent healthy food spending was determined by dividing the amount of money spent on healthy foods at the selected grocer during a month by the total amount spent on all food items at this grocer during that month. Secondary outcomes also examined shopping behavior at the selected grocer and included the average monthly percent unhealthy food spending (unhealthy food expenditures/total food expenditures), the average monthly percent healthy items (healthy food items/total food items), and the average monthly percent unhealthy food items (unhealthy food items/total food items).

### Member involvement

Vitality members were not involved in the research design or in the selection of outcome measures.

### Study intervention

After removing members who opted out, the remaining individuals were randomized to one of the six intervention arms that differed in the combination of financial incentive structures, weekly text messages, and monthly text messages (Table 1): Arm 1 (Usual Care): 10% cash back, no weekly text, standard monthly text; Arm 2: 10% cash back, generic weekly text, standard monthly text; Arm 3: 10% cash back, personalized weekly text, standard monthly text; Arm 4: 25% cash back, personalized weekly text, standard monthly text; Arm 5: 10 + 15%NET cash back, personalized weekly text, standard monthly text; and, Arm 6: 10 + 15%NET cash back, personalized weekly text, unbundled monthly text.

**Table 1:**
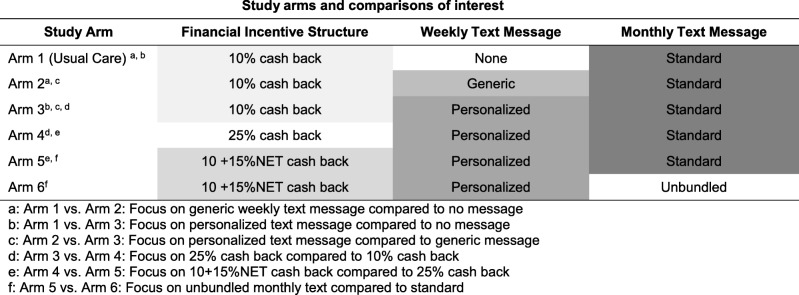
Study arms and comparisons of interest

The tested financial incentive structures were: 1) remaining at 10% cash back, 2) an increase to 25% cash back, or 3) 10 + 15%NET cash back. In the 10 + 15%NET participants could earn additional cash back above the baseline 10% level, but would also experience a financial penalty for unhealthy food purchases. This additional cash back was 15% of the difference between participants’ monthly healthy and unhealthy purchases. The rationale for this 10 + 15%NET structure was twofold. First, participants would only receive additional cash back (above the 10%) if they spent more on healthy foods than on unhealthy foods and would maximize their monthly cash back by purchasing no unhealthy foods. Second, no participant would earn less than the baseline 10% cash back amount (Vitality requirement to ensure there was no penalty to members for participating in the study).

The tested weekly text messaging strategies were: 1) no message (usual care for HF members), 2) a generic weekly text message (general information on the HF program and healthy eating habits, with different messages for each week of intervention), and 3) a personalized weekly text message (detailed individual feedback including the monetary and item breakdown of healthy vs. unhealthy purchases made at the selected grocer during the prior week) (Table 2). All weekly text messages were sent on Fridays. We were not able to confirm that these messages were opened and read.

**Table 2:** Examples of Weekly and Monthly Text Messages

Weekly Text Messages	
Generic Weekly Text Examples	
• *Discovery Vitality: The HealthyFood benefit makes it easier to purchase healthy foods which are essential to maintaining good health!*	
• *Discovery Vitality: Vitality’s HealthyFood benefit aims to make eating healthily easier and more affordable.*	
• *Discovery Vitality: HealthyFood refers to a range of foods including vegetables, fruit, wholegrains, lean meats, fat-free dairy, legumes and healthy fats.*	
Personalized Weekly Text Example	
• *Discovery Vitality: Last week, R234.77 (11 items) of your food spend was HealthyFood and R266.65 (9 items) was unhealthy. Eat HealthyFood for great cash rewards!*	
Monthly Text Messages	
Standard Monthly Text Message Example	
• *Discovery Vitality: The healthy foods you purchased have given you a R236.98 cashback. Keep it up! Visit* *discovery.co.za* *for useful nutrition and health tips.*	
Unbundled Monthly Text Message Example:	
• *Discovery Vitality: You earned a R236.98 HealthyFood cashback. You could have earned an additional R100.81 had you purchased no unhealthy foods. Visit* *discovery.co.za* *for useful nutrition and health tips.*	

In the HF program, all members receive a monthly text message informing them of their monthly cash back deposit. Given the introduction of the 10 + 15%NET incentive structure, which included a penalty for unhealthy purchases, we added a new monthly text messaging strategy to highlight financial losses incurred due to unhealthy food purchases (referred to as the unbundled monthly text message) (Table 2).

The weekly text messages began in November 2015 (referred to as start of the Partial Intervention). The Full Intervention (financial incentive changes, weekly text, and monthly text) was delayed due to unexpected Vitality operational issues and began in January 2016 and lasted through July 2016.

### Statistical analysis

Participant characteristics and historical shopping patterns were summarized by study arm. We had six pre-specified pairwise comparisons of interest, each chosen to isolate different financial incentive or text messaging strategy comparisons (Table 1). For example, comparing Arm 1 vs. Arm 2 highlighted the difference between no weekly message and the generic weekly message, while comparing Arm 2 vs. Arm 3 isolated the potential difference between the generic vs. personalized weekly text messages. Given this number of planned comparisons, we applied the Holm-Bonferroni procedure to correct for multiple comparisons. This approach uses a step-wise approach to compare the k-th smallest *p*-value out of the six pre-specified comparisons to a threshold of 0.05/k for significance, starting with the smallest p-value and stopping the first time the threshold for significance is not met, at which point no further hypotheses are declared significant [13]. We powered the study using the initial p-value threshold for statistical significance of 0.05/6 = 0.008. With 450 per arm, we had 80% power to detect an absolute difference of 3.7% (e.g., 29.4% versus 33.1%) in the percent healthy spending between study arms, assuming the control mean (standard deviation) of 29.4% (15.7%).

We used the Wilcoxon rank-sum test to assess between-arm differences in the primary and secondary outcomes. Some participants did not shop at the selected grocer during each month of the Full Intervention period. To handle these missing monthly shopping data points most conservatively, we assumed a worst-case scenario. Missing percent monthly healthy spending or percent monthly healthy items were designated as 0%; the rationale was that a percent healthy spending or items of 0% reflected a complete lack of engagement with the HF program, while a non-0 % healthy spending reflected the degree of engagement. Inversely, when monthly shopping data was missing, the percent unhealthy spending or unhealthy items were designated as 100% unhealthy. As an alternative approach to handling these missing shopping data points, we applied the multiple imputation using chained equations (MICE) algorithm [14, 15]. The imputation approach estimated the joint distribution for the participants’ shopping pattern for the 12 months prior to the intervention period, monthly shopping during the intervention period itself, and the shopping pattern from the month following the intervention, as well as available participant demographic variables (age, gender, household size, length of HF membership, geographic region), baseline transaction count (number of shopping trips at selected grocer during month before intervention), and assigned study arm, in order to impute the missing shopping data. Differences between arms were averaged across 25 imputations and the variance for inference was calculated using Rubin’s rule [14].

#### Additional analyses

We conducted two additional pre-specified analyses. First, to examine longitudinal trends in the primary and secondary outcomes, we applied a linear mixed model to the monthly data. The model included fixed effects for study arm, a linear time trend and an interaction term between study arm and time trend, as well as a random effect for participant. We tested the differences in the study arm and time trend interaction terms to determine whether slopes differed between study arms. Second, we repeated the main analysis of the primary and secondary outcomes using multivariable regression models to control for baseline participant demographics and their baseline shopping behavior (during the 12 months prior to the intervention). We also conducted an additional post-hoc (not pre-specified) analysis to examine higher-level themes across the study arms. We compared the primary and secondary outcomes between those getting the lower (10%) incentive amount (Arms 1–3) vs. those getting the higher (10 + 15%NET or 25%) incentive amount (Arms 4–6) and between those who received no/generic weekly message (Arms 1 and 2) vs. those who received a personalized weekly message (Arms 3–6). All analyses were conducted in Stata 12.1 (StataCorp).

## Results

**Fig. 1:**
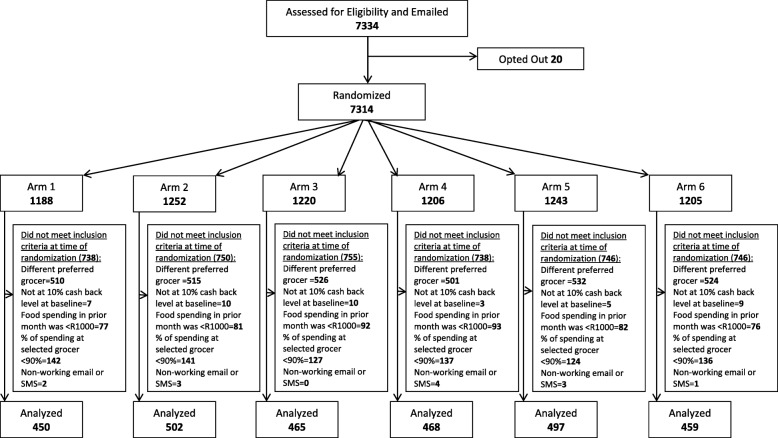
CONSORT Diagram. Flow diagram of member recruitment, randomization, inclusion criteria, and analytic sample

Figure 1 shows the Consolidated Standards of Reporting Trials (CONSORT) diagram for the study. After removing the 20 individuals who opted out of participating, the remaining 7314 people were randomized to one of the six study arms. Due to a technical error in the identification process, this randomized cohort erroneously included 3108 members who did not have the selected grocer as their preferred shopping partner and an additional 1365 people who did not meet at least one of the other pre-specified inclusion criteria. This error was discovered following data collection. To adhere to our original inclusion and exclusion criteria, these non-eligible participants were removed from the final analyzed cohort (reasons for exclusion detailed in Fig. 1). The number of people removed from each arm due to this error did not differ (*p* = 0.77) and those removed did not differ in age (*p* = 0.47), family size (*p* = 0.17), or length of Vitality membership (*p* = 0.94) from the final analytic cohort (Additional file 1: Table S1). While the proportions of women (35.3% of those removed vs. 46.5% of the final cohort, *p* < 0.01) and the people living in Gauteng region (58.2% of those removed vs. 65.7% of the final cohort, *p* < 0.01) differed between the removed and final populations, these differences are difficult to interpret as the gender is that of the primary Vitality member (not the person doing the grocery shopping) and the concentration of the selected grocer’s store locations varies by region. The final analytic sample consisted of 2841 eligible participants.

The age, gender, household size, and geographic region distribution of members were similar between all study arms (Table 3). In the overall analytic cohort, 46.5% of participants were female, the mean age was 47.8 years (11.8), and the mean household size was 2.9 (1.2) members. The participants in the six arms were similar in their baseline shopping behaviors during the 12 months prior to the intervention (Table 3). For the entire analyzed cohort, the average number of monthly shopping transactions at the selected grocer prior to the intervention was 8.0 (4.2), and the baseline average healthy food spending amount was R883.4 (R541.9), approximately £50. At baseline, the percent monthly healthy and unhealthy spending for the entire cohort were 27.7% (10.7%) and 17.5% (7.4%), respectively (Table 3). The baseline percent healthy spending and items are higher than those in the intervention period, and the unhealthy spending and items lower, reflecting our aforementioned conservative approach to missing data (i.e., missing = 0% healthy and 100% unhealthy).

**Table 3:** Participant demographic and baseline shopping characteristics

Study Arm	1	2	3	4	5	6	Overall
N	450	502	465	468	497	459	2841
Intervention Components							
*Cash Back %*	*10%*	*10%*	*10%*	*25%*	*10 + 15%NET*	*10 + 15%NET*	
*Weekly Text Message*	*None*	*Generic*	*Personalized*	*Personalized*	*Personalized*	*Personalized*	
*Monthly Text Message*	*Standard*	*Standard*	*Standard*	*Standard*	*Standard*	*Unbundled*	
Characteristics							
Female (%)^a^	47.1	44.8	47.3	44.2	49.3	46.2	46.5
Age, mean (SD)	47.9 (11.8)	48.3 (11.7)	47.0 (11.6)	47.4 (11.6)	47.7 (11.9)	48.5 (12.0)	47.8 (11.8)
Household Size, mean (SD)	3.0 (1.3)	2.9 (1.3)	3.0 (1.2)	3.0 (1.2)	2.9 (1.2)	2.9 (1.3)	2.9 (1.2)
Vitality membership, mean weeks (SD)	110.5 (56.9)	110.1 (56.2)	111.7 (58.3)	107.3 (55.6)	112.2 (58.2)	112.4 (57.8)	110.7 (57.1)
Region (%)							
Gauteng	64.2	65.5	64.9	69.7	66.2	63.4	65.7
Kwazulu-Natal	8.7	8.2	9.9	7.5	10.3	9.4	9.0
Western Cape	19.1	20.3	16.3	15.4	16.7	18.5	17.7
Other	8.0	6.0	8.8	7.5	6.8	8.7	7.6
Shopping trips/month, mean (SD)	7.9 (4.4)	7.9 (4.1)	8.1 (4.0)	7.7 (4.3)	7.9 (4.1)	8.3 (4.2)	8.0 (4.2)
Healthy Spending, mean Rand (SD)	895.0 (548.9)	923.4 (608.9)	851.8 (511.9)	843.5 (506.0)	878.9 (540.5)	906.1 (521.4)	883.4 (541.9)
Unhealthy Spending, mean (SD)	558.3 (347.1)	539.0 (302.2)	549.0 (326.1)	515.0 (303.6)	532.0 (313.0)	526.6 (332.4)	536.5 (320.6)
Neutral Spending, mean (SD)	1823.5 (963.5)	1760.5 (881.9)	1818.8 (944.6)	1727.3 (888.4)	1736.5 (930.6)	1786.6 (900.5)	1774.6 (918.1)
# Healthy Items, mean (SD)	36.1 (21.2)	37.5 (23.7)	34.9 (20.5)	34.8 (21.0)	35.9 (21.6)	37.1 (20.6)	36.1 (21.5)
# Unhealthy Items, mean (SD)	26.4 (18.4)	24.6 (14.9)	25.2 (15.8)	23.7 (15.1)	24.2 (15.4)	24.1 (16.4)	24.7 (16.0)
# Neutral Items, mean (SD)	53.7 (28.8)	51.8 (26.2)	52.8 (27.0)	50.2 (25.7)	50.7 (26.1)	52.2 (27.0)	51.9 (26.8)
% Healthy Spending, mean (SD)	27.5 (10.4)	28.3 (11.8)	26.6 (9.7)	27.7 (10.2)	27.9 (10.5)	28.5 (11.4)	27.7 (10.7)
% Unhealthy Spending, mean (SD)	17.8 (7.6)	17.7 (7.3)	17.7 (7.6)	17.3 (6.8)	17.7 (7.5)	16.9 (7.5)	17.5 (7.4)
% Healthy Items, mean (SD)	31.4 (11.2)	32.5 (12.7)	30.8 (10.7)	32.1 (11.3)	32.2 (11.6)	33.1 (12.8)	32.0 (11.8)
% Unhealthy Items, mean (SD)	22.6 (9.5)	22.1 (9.3)	22.5 (9.4)	21.9 (9.0)	22.1 (9.2)	21.1 (9.3)	22.1 (9.3)

NOTE: Shopping characteristics are monthly averages across the 12 months prior to intervention

^a^This gender breakdown reflects the gender of the primary Vitality member, not necessarily the family member doing the shopping

During the intervention period, 2351 participants (82.5%) had complete shopping data (at least one transaction at selected grocer during each month of the Full Intervention), and 209 participants (7.4%) were missing only one month of shopping data. Only 67 participants (2.4%) were missing shopping data for all the months. The average percent monthly healthy spending (reflecting program engagement and level of spending i.e., the missing monthly shopping data = 0% healthy spending) ranged from 24.8% (11.4%) in Arm 1 to 26.8% (12.9%) in Arm 2 (Table 4). This difference between Arm 1 and Arm 2 was the largest in magnitude, but the associated *p*-value of 0.093 did not surpass the Holm-Bonferroni corrected threshold of 0.008

**Table 4:** Average monthly spending and items by Arm (mean [SD])

Study Arm	1	2	3	4	5	6						
Intervention Components												
*Cash Back %*	*10%*	*10%*	*10%*	*25%*	*10 + 15%NET*	*10 + 15%NET*						
*Weekly Text Message*	*None*	*Generic*	*Personalized*	*Personalized*	*Personalized*	*Personalized*	*p*-values					
*Monthly Text Message*	*Standard*	*Standard*	*Standard*	*Standard*	*Standard*	*Unbundled*	1v 2	1 v 3	2 v 3	3 v 4	4 v 5	5 v 6
Outcomes												
% Healthy Spending	24.8 (11.4)	26.8 (12.9)	24.9 (11.7)	25.7 (11.5)	25.9 (12.7)	26.5 (13.0)	0.093	0.652	0.032	0.213	0.880	0.572
% Unhealthy Spending	24.4 (20.1)	21.7 (17.2)	22.4 (17.7)	21.7 (17.1)	23.9 (19.9)	22.3 (19.1)	0.031	0.121	0.547	0.514	0.121	0.039
% Healthy Items	27.8 (12.5)	30.2 (13.9)	28.4 (12.7)	29.5 (12.8)	28.9 (13.6)	30.0 (14.5)	0.063	0.971	0.067	0.211	0.545	0.365
% Unhealthy Items	29.4 (19.8)	26.6 (17.5)	27.2 (17.7)	26.3 (17.2)	28.5 (19.8)	26.9 (19.2)	0.028	0.106	0.561	0.389	0.148	0.068

needed to be considered statistically significant. Repeating the examination of average percent monthly healthy spending using imputed missing shopping data yielded similar results, with no statistically significant differences between any of the arms (Additional file 1: Table S2).

No statistically significant between-arm differences were noted for any of the secondary outcomes as well (Table 4). The analysis using imputed missing shopping data also yielded similar results for the secondary outcomes, with no statistically significant between-arm differences (Additional file 1: Table S2).

**Fig. 2:**
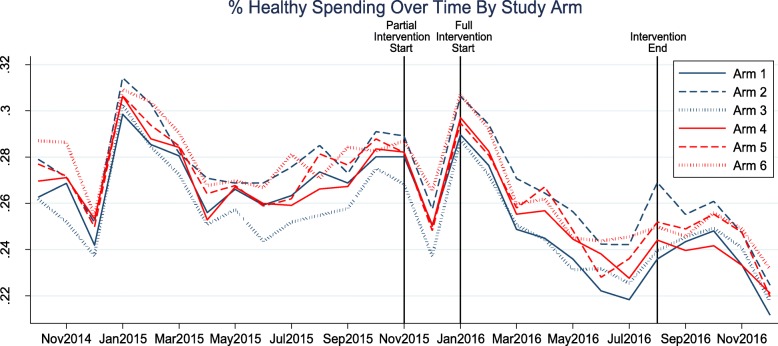
Percent healthy spending over time by Study Arm. Arm 1 (Usual care): 10% cash back, no weekly text, standard monthly text; Arm 2: 10% cash back, generic weekly text, standard monthly text; Arm 3: 10% cash back, personalized weekly text, standard monthly text; Arm 4: 25% cash back, personalized weekly text, standard monthly text; Arm 5: 10 + 15%NET, personalized weekly text, standard monthly text; Arm 6: 10 + 15%NET, personalized weekly text, unbundled monthly text

In the additional analyses, we examined the trend in the primary and secondary outcomes using the monthly data during the Full Intervention period. A sharp peak in the holiday months (December–January) followed by a downward trend was noted across all study arms (Fig. 2). As in the primary analysis, the widest gap between trend lines emerged for Arm 1 and Arm 2; however, the downward slopes of all arms’ trends were approximately 0.01, representing a decrease of 1% in healthy spending for each month that passed (Additional file 1: Table S3). None of the between-arm slopes differed significantly. Finally, when controlling for baseline participant demographics and shopping behaviors in a multiple regression, we found Arm 2 had a 1.3% higher average percent monthly healthy spending than Arm 1, but the associated *p*-value of 0.017 was not significant after Holm-Bonferroni correction (Additional file 1: Table S3). In this regression analysis, Arm 2 had a 1.6% higher average percent monthly healthy items than Arm 1, but this difference was also not statistically significant (*p* = 0.014) (Additional file 1: Table S3). Finally, no differences in the shopping outcomes were noted when grouping the arms by higher-level themes (lower vs. higher incentive, no/generic weekly message vs. personalized weekly message) (Additional file 1: Table S4 and Additional file 1: Table S5).

## Discussion

In this RCT of adult members of an insurer-grocery store partnered healthy food promotion program, none of the tested financial incentive and text messaging combinations differentially affected the examined shopping outcomes, monthly healthy and unhealthy spending and item counts.

The absence of any appreciable change in food purchasing among those whose cash back was more than doubled (increase from 10 to 25%) and those who experienced a new financial disincentive for unhealthy foods (10 + 15%NET) is noteworthy. Past work has repeatedly demonstrated the power of financial incentives to influence health-related behaviors, including fruit and vegetable purchases, weight loss, smoking cessation, and vaccination rates [7, 16–18]. The effect of financial disincentives on health-related behaviors has also been established in the literature; observational and simulation studies demonstrate the effectiveness of taxes on decreasing the purchasing of unhealthy items, including sugar sweetened beverages, less-healthy foods, alcohol, and cigarettes [19–26].

There are several possible reasons why the tested financial incentive structures did not affect members’ purchasing behaviors. First, the absence of any effect may reflect the temporal separation between the shopping and the cash back payments. To overcome the present bias that can contribute to unhealthy food choices, as well as to leverage the regret aversion that can also influence choices, more immediate savings or penalties, experienced at the time of purchase, may be more effective than what was implemented in this study. Our original study design called for immediate feedback after each shopping trip; unfortunately, lags in the processing of the shopping data made this design unworkable. Second, we focused on HF members who were still at the baseline 10% cash back level. This lower engagement with HF may reflect lower cost sensitivity since more cost sensitive individuals would likely have completed the simple tasks necessary to get to the highest cash back level of 25%. In a cost-insensitive population, the monetary difference between the 10 and 25% cash back levels and the potential financial losses in the 10 + 15%NET Arms may have been of insufficient size to shift purchasing behaviors. Of note, the participants in the 10 + 15%NET Arms (Arms 5 and 6) were still getting 10% cash back regardless of any unhealthy food purchases. The effect of this incentive structure might have been different if unhealthy food choices had resulted in participants’ cash back amount being below their baseline 10% level (cash back below 10% was not tested because Vitality did not want to penalize members for study participation). Finally, the lack of observed effect of the financial incentives and disincentives may reflect a different valuation system. It is possible that individuals who are consuming unhealthy foods derive considerable utility from doing so, and this utility may be equally or more valuable than the financial gains and losses resulting from their shopping choices.

Given the literature supporting the value of tailored, individualized nutritional feedback over generic feedback, the absence of differences between Arm 1 (no weekly message) and Arm 3 (personalized weekly message) and between the no/generic weekly message arms (Arms 1 and 2) and the personalized message arms (Arms 3–6) were unexpected [9, 10]. One possibility is that the personalized weekly text feedback was confusing, not motivating, contained the “wrong” information, or required too much mental accounting. To maximize the potential of tailored messaging strategies, future work is needed to improve message quality by identifying the ideal message frequency, timing, and content to optimally support engagement in programs like HF. While only noted in the adjusted regression analyses and bordering on statistical significance, there was a suggestion of differences in the monthly percent healthy food spending and monthly percent healthy food items between Arm 1 (no weekly message) and Arm 2 (generic weekly message). One possible explanation for this unanticipated, possible difference was that any message, even a generic one, served as a reminder for HF members to take advantage of the program.

The study design had several limitations. First, the generalizability of the study findings to other contexts was limited by the current uniqueness of Vitality and the HF program, as well as the limited demographic information available on participants. While we had no information on participants’ income, a likely contributor to their sensitivity to financial incentives and penalties, we do know that, in South Africa, having private insurance, like Discovery Vitality, is associated with higher income, suggesting this population may be relatively cost insensitive. Second, the weekly text messages only reflected participants’ shopping at the selected grocer. Members likely shopped at multiple stores or only purchased certain foods at certain stores. Given this, the weekly text messages may not have accurately described their shopping behaviors, thereby diminishing the potential impact of this personalized feedback. Third, the classification of foods as healthy, unhealthy, and neutral is an inherently noisy signal in this context for many reasons. While healthy foods are labeled on store shelves, unhealthy foods are not, limiting participants’ ability to actively avoid them. Further, there are no clear guidelines regarding the ideal make up of a grocery cart (i.e., what is the ideal percent healthy and unhealthy?) and no data on what a “normal” percent healthy basket is for different populations (i.e., were enrolled participants already at the high end of basket healthiness?). Fourth, the incentive structure changes, particularly the 10 + 15%NET, may have been confusing for participants or entirely missed by participants, limiting any effect of these changes on participants’ food purchasing behaviors. Unfortunately, we were not able to assess members’ awareness or understanding of any changes to their benefit design. Fifth, we were unable to determine if the weekly and monthly text messages were opened and read by participants.

The study design reveals how existing programs can become laboratories to study approaches to improve health. This study was conducted in the same setting in which interventions would be potentially be implemented. And, while this pragmatic design led to some unexpected challenges (e.g., delay in start of the Full Intervention, protocol deviation during recruitment that reduced sample size and limited statistical power) and made certain design features unfeasible (e.g., immediate feedback on shopping, cash back below 10%), it also offered the chance to assess evidence-based interventions in a real-world setting.

## Conclusions

The results of this RCT suggest that merely changing the amount of a delayed financial incentive or introducing a small financial penalty for unhealthy food choices are insufficient when trying to shift food purchasing behaviors among “low utilizing” members of a healthy food promotion program. While it is possible that improving peoples’ dietary habits merely requires larger and more immediate financial benefits or consequences or better messaging, it is more likely that this complex, diverse, multi-dimensional problem requires equally multi-dimensional and tailored solutions. The United Kingdom Behavioral Insights team’s EAST framework emphasizes that interventions to change behaviors need to be easy, attractive, social, and timely [27]. Building on this framework and expanding beyond financially-centered motivators, effective interventions to promote healthier diets may require a combination of creative strategies, including changing default options (e.g., a default healthy basket for online grocery shoppers), creating choice architecture environments that support healthier choices (e.g., more salient in-store signage and displays), leveraging intrinsic motivators (e.g., aligning HF incentives with participant’s own goals), and utilizing technology-based tools (e.g., new Vitality app that allows users to scan foods and quickly identify healthier alternatives). Given the importance of a healthy diet for chronic disease prevention and management, innovative strategies to support optimal food choices at the point of purchase will remain a focus for health behavior change researchers and public health practitioners. Partnerships with food retailers and insurers hold great potential to offer real-world opportunities for implementing and evaluating these novel strategies.

## Additional file

Additional file 1: This document includes 5 tables describing results of all additional analyses described in the test. **Table S1**: Comparing demographic characteristics of those removed from cohort due to protocol deviation during recruitment. **Table S2**: Average monthly shopping spending and items by Arm with missing data imputed (mean [SD]). **Table S3**: Results of planned additional analyses, time trend and multivariable regression. **Table S4**: Average monthly spending and items by incentive level (mean [SD]). **Table S5**: Average monthly spending and items by weekly message type (mean [SD]) (DOCX 25 kb)

## Abbreviations

CONSORT: Consolidated Standards of Reporting Trials
HF: Healthy food program
R: South African Rand
RCT: Randomized controlled trial
SD: Standard deviation
