# Supplementary material for: Use of financial incentives and text message feedback to increase healthy food purchases in a grocery store cash back program: a randomized controlled trial
Source: BMC Public Health. 2019 May 31;19:674. doi: 10.1186/s12889-019-6936-5 (PMC6544953; doi:10.1186/s12889-019-6936-5)
Supplement: Supplementary file 1 — This document includes 5 tables describing results of all additional analyses described in the test. Table S1: Comparing demographic characteristics of those removed from cohort due to protocol deviation during recruitment. Table S2: Average monthly shopping spending and items by Arm with missing data imputed (mean [SD]). Table S3: Results of planned additional analyses, time trend and multivariable regression. Table S4: Average monthly spending and items by incentive level (mean [SD]). Table S5: Average monthly spending and items by weekly message type (mean [SD]) (DOCX 25 kb) [file 12889_2019_6936_MOESM1_ESM.docx]

| **Supplemental Table 1. Comparing demographic characteristics of those removed from cohort due to protocol deviation during recruitment** | | | |
| --- | --- | --- | --- |
|  | **Removed from Cohort**  **(N = 4473)** | **Final Analytic Cohort**  **(N = 2841)** | **p-value** |
| Female (%) | 35.3 | 46.5 | <0.01 |
| Age, mean (SD) | 48.3 (11.9) | 47.8 (11.8) | 0.47 |
| Household Size, mean (SD) | 3.0 (1.3) | 2.9 (1.2) | 0.17 |
| Vitality membership, mean weeks (SD) | 110.7 (57.2) | 110.7 (57.1) | 0.94 |
| Region (%) |  |  | <0.01 |
| Gauteng | 58.2 | 65.7 |  |
| Kwazulu-Natal | 9.4 | 9 |  |
| Western Cape | 23.8 | 17.7 |  |
| Other | 8.6 | 7.6 |  |

| **Supplemental Table 2. Average monthly shopping spending and items by Arm with missing data imputed (mean[SD])** | | | | | | | | | | | | |
| --- | --- | --- | --- | --- | --- | --- | --- | --- | --- | --- | --- | --- |
| **Study Arm** | **1** | **2** | **3** | **4** | **5** | **6** |  |  |  |  |  |  |
| **Intervention Components** |  |  |  |  |  |  |  |  |  |  |  |  |
| *Cash-Back %* | *10* | *10* | *10* | *25* | *10+ 15NET* | *10 + 15NET* |  |  |  |  |  |  |
| *Weekly Text Message* | *None* | *Generic* | *Personalized* | *Personalized* | *Personalized* | *Personalized* | **p-values** | | | | | |
| *Monthly Text Message* | *Standard* | *Standard* | *Standard* | *Standard* | *Standard* | *Unbundled* | 1 v 2 | 1 v 3 | 2 v 3 | 3 v 4 | 4 v 5 | 5 v 6 |
| **Outcomes** |  |  |  |  |  |  |  |  |  |  |  |  |
| % Healthy Spending | 27.1 (10.4) | 28.4 (12.3) | 26.6 (11.4) | 27.6 (11.2) | 28.0 (11.7) | 28.4 (12.1) | 0.080 | 0.524 | 0.022 | 0.182 | 0.623 | 0.639 |
| % Unhealthy Spending | 17.4 (7.8) | 16.8 (7.9) | 17.0 (8.3) | 16.2 (7.3) | 17.1 (7.9) | 16.3 (7.6) | 0.215 | 0.407 | 0.715 | 0.131 | 0.073 | 0.097 |
| % Healthy Items | 30.4 (11.2) | 32.0 (13.1) | 30.3 (12.2) | 31.7 (12.2) | 31.3 (12.4) | 32.2 (13.3) | 0.051 | 0.917 | 0.045 | 0.091 | 0.611 | 0.274 |
| % Unhealthy Items | 22.9 (10.0) | 22.0 (10.2) | 22.2 (10.2) | 21.2 (9.5) | 22.2 (10.1) | 21.3 (10.0) | 0.212 | 0.316 | 0.821 | 0.116 | 0.110 | 0.153 |

| **Supplemental Table 3. Results of planned additional analyses, time trend and multivariable regression** | | | | | | | | | | | | |
| --- | --- | --- | --- | --- | --- | --- | --- | --- | --- | --- | --- | --- |
| **Study Arm** | **1** | **2** | **3** | **4** | **5** | **6** |  |  |  |  |  |  |
| **Intervention Components** |  |  |  |  |  |  |  |  |  |  |  |  |
| *Cash-Back Level* | *10%* | *10%* | *10%* | *25%* | *10 + 15NET* | *10 + 15NET* |  |  |  |  |  |  |
| *Weekly Text Message* | *None* | *Generic* | *Personalized* | *Personalized* | *Personalized* | *Personalized* | **p-values** | | | | | |
| *Monthly Text Message* | *Standard* | *Standard* | *Standard* | *Standard* | *Standard* | *Unbundled* | 1 v 2 | 1 v 3 | 2 v 3 | 3 v 4 | 4 v 5 | 5 v 6 |
| **Linear Time Trend (Slope)*** |  |  |  |  |  |  |  |  |  |  |  |  |
| % Healthy Spending | -1.2 | -1.1 | -1.0 | -1.1 | -1.0 | -1.1 | 0.469 | 0.164 | 0.484 | 0.547 | 0.633 | 0.876 |
| % Unhealthy Spending | 1.2 | 1.0 | 0.8 | 1.2 | 1.3 | 1.1 | 0.257 | 0.068 | 0.465 | 0.080 | 0.443 | 0.304 |
| % Healthy Items | -1.1 | -0.9 | -0.8 | -0.9 | -1.0 | -1.0 | 0.257 | 0.049 | 0.377 | 0.336 | 0.775 | 0.675 |
| % Unhealthy Items | 1.3 | 1.0 | 0.9 | 1.2 | 1.4 | 1.2 | 0.147 | 0.064 | 0.659 | 0.119 | 0.281 | 0.180 |
| **Multivariate Regression****  **(β, %/month)** |  |  |  |  |  |  |  |  |  |  |  |  |
| % Healthy Spending | Referent | 1.3 | 0.9 | 0.9 | 0.7 | 0.8 | 0.017 | 0.122 | 0.418 | 0.977 | 0.797 | 0.820 |
| % Unhealthy Spending | Referent | -2.4 | -2.3 | -2.7 | -0.3 | -1.7 | 0.042 | 0.059 | 0.914 | 0.701 | 0.038 | 0.222 |
| % Healthy Items | Referent | 1.6 | 1.3 | 1.7 | 0.7 | 1.3 | 0.014 | 0.038 | 0.727 | 0.638 | 0.124 | 0.350 |
| % Unhealthy Items | Referent | -2.3 | -2.6 | -3.1 | -0.6 | -2.0 | 0.043 | 0.027 | 0.814 | 0.672 | 0.029 | 0.236 |
| * Model included fixed effects for study arm, a linear time trend and an interaction term between study arm and time trend, as well as a random effect for participant  ** Model included participant demographics and their baseline shopping behavior during the 12 months prior to the intervention | | | | | | | | | | | | |

| **Supplemental Table 4. Average monthly spending and items by incentive level (mean[SD])** | | | |
| --- | --- | --- | --- |
| **Study Arm** | **Lower incentive** | **Higher incentive** |  |
| *Included Arms* | *1, 2, 3* | *3,4,5* |  |
| **Outcomes** |  |  | p-value |
| % Healthy Spending | 0.255 (0.121) | 0.260 (0.124) | 0.265 |
| % Unhealthy Spending | 0.228 (0.183) | 0.227 (0.188) | 0.308 |
| % Healthy Items | 0.288 (0.131) | 0.295 (0.136) | 0.346 |
| % Unhealthy Items | 0.277 (0.183) | 0.273 (0.188) | 0.187 |

| **Supplemental Table 5. Average monthly spending and items by weekly message type (mean[SD])** | | | |
| --- | --- | --- | --- |
| **Study Arm** | **no/generic message** | **personalized message** |  |
| *Included Arms* | *1, 2, 3, 4* | *4,5* |  |
| **Outcomes** |  |  | p-value |
| % Healthy Spending | 0.256 (0.119) | 0.262 (0.128) | 0.244 |
| % Unhealthy Spending | 0.225 (0.180) | 0.232 (0.196) | 0.764 |
| % Healthy Items | 0.290 (0.130) | 0.294 (0.140) | 0.598 |
| % Unhealthy Items | 0.273 (0.181) | 0.278 (0.195) | 0.631 |
